# Genome-wide characterization of simple sequence repeats in cucumber (*Cucumis sativus *L.)

**DOI:** 10.1186/1471-2164-11-569

**Published:** 2010-10-15

**Authors:** Pablo F Cavagnaro, Douglas A Senalik, Luming Yang, Philipp W Simon, Timothy T Harkins, Chinnappa D Kodira, Sanwen Huang, Yiqun Weng

**Affiliations:** 1 Horticulture Department, University of Wisconsin, Madison, WI 53706, USA; 2 INTA La Consulta, and CONICET, Mendoza, Argentina; 3 USDA ARS Vegetable Crops Research Unit, Horticulture Department, University of Wisconsin, Madison, WI 53706, USA; 4 Roche Applied Sciences, Indianapolis, IN 46250, USA; 5 454 Life Sciences, Branford, CT 06405, USA; 6 Institute of Vegetables and Flowers, Chinese Academy of Agricultural Sciences, Beijing, 100081, China

## Abstract

**Background:**

Cucumber, *Cucumis sativus *L. is an important vegetable crop worldwide. Until very recently, cucumber genetic and genomic resources, especially molecular markers, have been very limited, impeding progress of cucumber breeding efforts. Microsatellites are short tandemly repeated DNA sequences, which are frequently favored as genetic markers due to their high level of polymorphism and codominant inheritance. Data from previously characterized genomes has shown that these repeats vary in frequency, motif sequence, and genomic location across taxa. During the last year, the genomes of two cucumber genotypes were sequenced including the Chinese fresh market type inbred line '9930' and the North American pickling type inbred line 'Gy14'. These sequences provide a powerful tool for developing markers in a large scale. In this study, we surveyed and characterized the distribution and frequency of perfect microsatellites in 203 Mbp assembled Gy14 DNA sequences, representing 55% of its nuclear genome, and in cucumber EST sequences. Similar analyses were performed in genomic and EST data from seven other plant species, and the results were compared with those of cucumber.

**Results:**

A total of 112,073 perfect repeats were detected in the Gy14 cucumber genome sequence, accounting for 0.9% of the assembled Gy14 genome, with an overall density of 551.9 SSRs/Mbp. While tetranucleotides were the most frequent microsatellites in genomic DNA sequence, dinucleotide repeats, which had more repeat units than any other SSR type, had the highest cumulative sequence length. Coding regions (ESTs) of the cucumber genome had fewer microsatellites compared to its genomic sequence, with trinucleotides predominating in EST sequences. AAG was the most frequent repeat in cucumber ESTs. Overall, AT-rich motifs prevailed in both genomic and EST data. Compared to the other species examined, cucumber genomic sequence had the highest density of SSRs (although comparable to the density of poplar, grapevine and rice), and was richest in AT dinucleotides. Using an electronic PCR strategy, we investigated the polymorphism between 9930 and Gy14 at 1,006 SSR loci, and found unexpectedly high degree of polymorphism (48.3%) between the two genotypes. The level of polymorphism seems to be positively associated with the number of repeat units in the microsatellite. The *in silico *PCR results were validated empirically in 660 of the 1,006 SSR loci. In addition, primer sequences for more than 83,000 newly-discovered cucumber microsatellites, and their exact positions in the Gy14 genome assembly were made publicly available.

**Conclusions:**

The cucumber genome is rich in microsatellites; AT and AAG are the most abundant repeat motifs in genomic and EST sequences of cucumber, respectively. Considering all the species investigated, some commonalities were noted, especially within the monocot and dicot groups, although the distribution of motifs and the frequency of certain repeats were characteristic of the species examined. The large number of SSR markers developed from this study should be a significant contribution to the cucurbit research community.

## Background

Microsatellites or simple sequence repeats (SSRs) are short (1-6 bp) tandemly repeated DNA sequences found ubiquitously in the genomes of prokaryotic and eukaryotic organisms [1-3]. Because of their high mutation rate, which results in allelic changes of array length, these repeats represent a rich source of hypervariable codominant markers [4,5]. Thus, microsatellite markers have become extensively used in many research areas such as linkage mapping [6,7], population genetics [8], parentage analysis [9]), and phylogenetic or comparative genomics research [10,11]. Besides their usefulness as genetic markers, microsatellites are thought to play important roles in genome evolution, e.g., by creating genetic variability [12], in regulation of gene expression [13,14] thereby conditioning the development of some human genetic disorders [15,16], and in chromatin organization, DNA replication and cell cycle [17].

The availability and analysis of nearly complete genome sequences from many organisms has provided insight into the distribution, evolution and putative function of microsatellites [17]. Microsatellite density and distribution across the genome is unequal and seemingly non-random, as suggested by SSR data from humans [18], plants [19] and other eukaryotic organisms [3], with commonalities and differences observed across taxa. For example, tri- and hexanucleotide repeats generally prevail in protein-coding sequences, whereas taxon-specific variation in distribution of repeat motifs in different genomic fractions (for example, intergenic or protein-coding regions *vs. *introns) has also been reported [3,19]. The distribution of microsatellites in the genome has practical implications with regard to their use as molecular markers. For example, compared to microsatellites from non-transcribed DNAs, genic SSR markers have higher transferability among related species, which facilitates their use as anchor markers for comparative mapping studies [20]. On the other hand, genic microsatellites are under a higher selection pressure [3], so they may not provide sufficient polymorphism to discriminate between closely related germplasm. In this case, SSRs in noncoding regions may be a valuable complement.

The genus *Cucumis *(family Cucurbitaceae) includes two economically important vegetable crop species that are cultivated worldwide: cucumber (*C. sativus *L., 2n = 2*x *= 14) and melon (*C. melo *L., 2n = 2*x *= 24). Cucumber is believed to be of Asian origin, and was domesticated around 1,500 BC [21-24]. Two inter-fertile botanical varieties, the cultivated *C. sativus *var. *sativus *L. and the wild type *C. sativus *var. *hardwickii *(Royle) Alef., comprise the primary gene pool of cucumber. Due to its narrow genetic base, the intraspecific genetic diversity in cucumber as revealed in early studies with marker types such as isozymes, RFLPs (restriction fragment length polymorphisms), AFLPs (amplified fragment length polymorphisms) or RAPDs (randomly amplified polymorphic DNAs), is relatively low (3-12%) as compared to other *Cucumis *species [25-31], hindering the development of high-resolution genetic maps in cucumber. This has encouraged efforts towards the development of marker systems in cucumber that have higher polymorphism and are more user-friendly. Among them, microsatellite markers have been developed [32-34] and used for genetic mapping and marker-assisted selection studies [35,36]. However, large scale development of microsatellite markers were not realized until the whole genome sequence of cucumber was available [37]. Recently from cucumber whole genome sequence, Ren et al. (2009) designed ~ 2,100 SSR primer pairs, from which a cucumber linkage map with 995 SSRs was constructed, which was the largest set of microsatellites mapped to date in this species [38]. The usefulness of these cucumber microsatellite markers has already been demonstrated in several recent linkage mapping, diversity, as well as phylogenetic studies [39-42]. Despite such progresses, the number of robust, informative and user-friendly markers (e.g., SSRs) publicly available for cucumber is still insufficient for some applications, particularly considering the low intra-specific polymorphism level observed even with microsatellite markers (10-20%) [41,42]. The availability of a very large set of microsatellite markers distributed throughout the genome would facilitate the development of high-resolution maps (or rapid saturation of specific map regions), which are instrumental for applications like positional gene cloning and detailed comparative mapping, among others. Such molecular resources would benefit the cucumber research and breeding community.

Recent developments in high throughput DNA sequencing technologies, such as the Roche/454 FLX and Illumina's Solexa platforms [43] provide new opportunities to expedite molecular marker development. In the past year, the genomes of two cucumber genotypes have been sequenced; the Chinese fresh market type inbred line '9930' [37] (database is hosted at http://cucumber.genomics.org.cn/), and the North American pickling type inbred line 'Gy14' (genome and EST assembly are available at http://cucumber.vcru.wisc.edu/; genome annotation is available at http://www.phytozome.net/cucumber.php#A). In spite of the importance of microsatellite markers in many applications, systematic and genome-wide characterization of microsatellite sequences in the cucumber genome have not yet be conducted. In the present study, we have characterized the distribution and density of perfect microsatellites in ~ 203 Mbp of non-redundant DNA sequence from the nuclear genome of Gy14, as well as in cucumber EST sequence data. Comparative analysis using genomic and EST sequence datasets from other plant species was performed. In addition, primer sequences for more than 83,000 newly-discovered cucumber microsatellites, and their exact positions in the Gy14 genome assembly, were made publicly available.

## Results

We analyzed the distribution of perfect microsatellites with ≥3 repeat units and a minimum total length of 12 bp in ~ 203 Mbp of non-redundant cucumber genomic sequence, representing 55.3% of its 367 Mbp nuclear genome [44]. Repeats were first analyzed on a whole genome basis using genomic DNA sequence of cucumber inbred line Gy14 and other species (for comparison purposes). We then examined SSR frequencies in transcribed (EST) sequences, and compared them with their genomic sequence counterparts. For consistency in estimating repeat frequencies, the SSR motifs presented here represent all variants of both strands of the DNA sequence (e.g., AC also includes CA and the reverse complements GT and TG). Unless otherwise stated, microsatellite content in DNA sequences was expressed as 'number of SSRs per million base pairs' or as relative frequencies (%) within a particular dataset.

### Distribution of SSR types in genomic sequences

The content of perfect microsatellites in genomic sequences of cucumber and seven other plant species is summarized in Table 1. A total of 112,073 SSRs with perfect repeats were detected in the Gy14 whole genome sequence, which translated to an overall density across the genome of 551.9 SSRs/Mbp (i.e., one SSR every 1.8 kb of sequence, excluding mononucleotide SSRs). Surprisingly, cucumber had the highest microsatellite density among the species analyzed, comparable to that of poplar (508.3 SSR/Mbp), grapevine (506.9 SSR/Mbp) and rice (529.4 SSR/Mbp) (Table 1). Taken together, the total length of di- to octanucleotide repeats was estimated to account for about 0.9% of the assembled Gy14 cucumber genome (1% if mononucleotides were included; data not shown).

**Table 1:** Distribution of perfect microsatellites with ≥3 repeats and minimum 15 bp length in genomic and EST sequences of cucumber and selected plant species^#^

	Cucumber				Microsatellite density (SSR/Mbp)								
				
Sequence type	Count	Rel. freq. (%)	Mean repeat number	Density (SSR/Mb)	*Mt*	*Gm*	*Pt*	*At*	*Vv*	*Oz*	*Sb*	All species mean	***T-test *statistics (*T*, *P*)**^**§**^
SSR type													
**Genomic**													
Dinucleotide	29,651	26.5	9.6	146.0	61.5	74.6	132.6	78.7	117.2	100.1	51.6	95.3*	(2.57, 0.022)
Trinucleotide	28,645	25.6	4.9	141.1	97.0	**103.1**	132.2	**146.6**	115.8	**220.1**	**108.5**	133.0*	(2.27, 0.039)
Tetranucleotide	**33,348**	**29.8**	**3.2**	**164.2**	**102.8**	97.5	**144.9**	93.2	**171.3**	132.7	105.4	126.5*	(2.86, 0.013)
Pentanucleotide	11,038	9.8	3.2	54.4	40.0	27.2	62.1	32.0	58.0	45.8	22.5	42.8	n.s.
Hexanucleotide	6,290	5.6	3.3	31.0	14.4	16.8	26.9	13.6	29.3	27.4	27.5	23.4	n.s.
Heptanucleotide	2,337	2.1	3.3	11.5	2.5	7.1	7.3	5.8	12.4	2.5	1.9	6.3	n.s.
Octanucleotide	764	0.7	3.2	3.8	0.3	0.4	1.0	0.7	2.8	0.8	0.2	1.2	n.s.
Total/mean	**112,073**	**100**	**5.3**	**551.9**	**318.4**	**326.7**	**508.3**	**370.5**	**506.9**	**529.4**	**317.6**	**428.5**	n.s.
Total seq. (Mbp)^‡^				203.1	109.5	242.8	307.8	119.2	303.1	370.8	738.5		
GC content (%)				32.3	35.2	36.6	33.3	36.0	34.4	43.6	43.9		
**EST-clustered**^†^													
Dinucleotide	1,413/2,305	16.3/14.8	7.6/8.3	75.0/54.8	36.6	81.1	86.6	47.8	40.7	57.9	52.1	59.7	
Trinucleotide	**3,685/7,560**	**42.6/48.5**	**4.9/5.0**	**195.5/179.7**	**160.0**	**179.9**	**177.4**	**231.8**	**108.4**	**485.7**	**366.3**	**238.1**	
Tetranucleotide	1,892/2,916	21.9/18.7	3.2/3.3	100.4/69.3	74.5	81.2	91.6	50.9	53.3	107.3	125.5	85.6	
Pentanucleotide	807/978	9.3/6.3	3.3/3.4	42.8/23.3	25.9	27.5	30.2	12.4	17.4	41.6	44.1	30.2	
Hexanucleotide	635/1,519	7.3/9.7	3.3/3.4	33.7/36.1	25.9	29.2	34.1	13.4	22.1	44.6	56.5	32.4	
Heptanucleotide	162/230	1.9/1.5	3.3/3.3	8.6/5.5	1.7	3.6	4.0	0.9	4.2	1.9	1.4	3.3	
Octanucleotide	52/95	0.6/0.6	3.3/3.2	2.8/2.3	0.2	0.4	0.8	0.7	1.0	0.2	0.2	0.8	
Total/mean	**8,646/15,603**	**100/100**	**4.7/4.7**	**458.7/370.9**	**324.9**	**403.0**	**424.7**	**357.8**	**247.3**	**739.3**	**646.1**	**450.2**	
Total seq. (Mbp)^‡^				18.8/42.1	51.9	51.3	67.6	74.8	81.4	158.2	32.4		
GC content (%)				39.5/41.4	39.6	41.5	42.4	42.7	43.9	51.5	52.0		
**EST-bulked**													
Dinucleotide	3,515	12.9	7.5	55.2									
Trinucleotide	**12,151**	**44.6**	**4.8**	**190.9**									
Tetranucleotide	5,798	21.3	3.2	91.1									
Pentanucleotide	3,037	11.1	3.2	47.7									
Hexanucleotide	2,155	7.9	3.2	33.9									
Heptanucleotide	447	1.6	3.6	7.0									
Octanucleotide	155	0.6	3.2	2.4									
Total/mean	**27,258**	**100**	**4.5**	**428.2**									
Total seq. (Mbp)^‡^				63.7									
GC content (%)				40.3									

^# ^*Mt *= *Medicago truncatula*, *Gm = Glycine max*, *Pt *= *Populus trichocarpa*, *At *= *Arabidopsis thaliana*, *Vv *= *Vitis vinifera, Oz = Oryza sativa, Sb = Sorghum bicolor. *Data for highest frequencies and totals are denoted with boldface typed numbers. ^† ^Two clustered EST data sets from cucumber lines WI 1983 and Gy14 (WI 1983/Gy14) were analyzed. ^‡ ^Total length of sequences analyzed.* Value is significantly higher than its EST counterpart, at *P *≤ 0.05. n.s. Value not significantly different from its EST counterpart. ^**§ **^*T *= t-test statistic, *P *= probability value. Only SSRs with 3 or more repeat units and a minimum 12 bp were considered.

Tetranucleotides were the most common SSR type in cucumber genomic sequence representing nearly 30% of all SSRs, followed by di- (26.5%) and trinucleotides (25.6%) (Table 1). Hepta- and octanucleotides were the least frequent repeat types, together representing less than 3% of the total SSRs. The distribution of SSR types in cucumber was most similar to that of poplar and grapevine, which had comparable relative and absolute frequencies for each SSR type, and least similar to the distribution in rice and *Arabidopsis*, for which trinucleotides were by far the most frequent repeat type (Table 1). In both monocot species (rice and sorghum) trinucleotides predominated whereas tetranucleotides were more abundant in most (4 out of 6) of the dicot species analyzed. Other than this observation, no major differences were evident between monocots and dicots, and the range of variation in repeat density of both groups largely overlapped for most SSR types.

**Figure 1:**
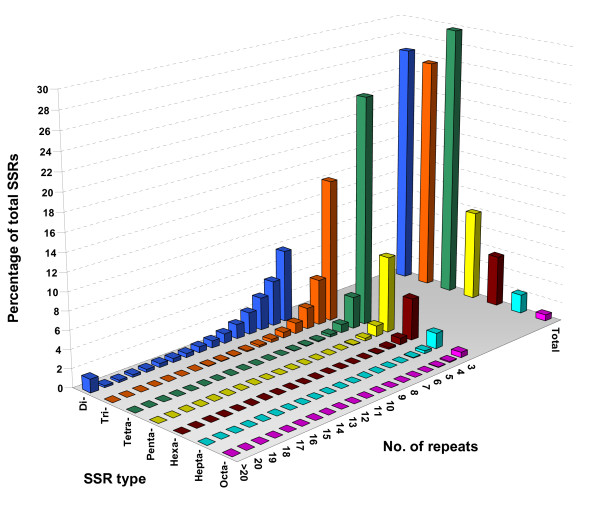
**Relative frequency (%) of SSR types, by number of repeats, in the cucumber genome**. The graph was based on a total of N = 112,073 SSRs detected in 203 Mbp non-redundant genomic DNA sequence of the Gy14 genome.

We examined the distribution of cucumber microsatellites with regard to the number of repeat units (Figure 1). For all SSR classes, microsatellite frequency decreased as the number of repeat units increased. However, the rate of this change was more gradual in dinucleotides than in longer repeat types, with tetra- to octanucleotides showing the most dramatic reduction in frequency as they increased repeat units. As a consequence, the mean number of repeat units in dinucleotides (9.6) was nearly twice as much as the number of repeat units in trinucleotides (4.9) and it was three times higher than in tetra- to octanucleotides (3.2 - 3.3) (Table 1). Thus, although tetranucleotides (164.2 SSRs/Mbp) occurred more frequently than dinucleotides (146.0 SSRs/Mbp) in the Gy14 assembled genome, the dinucleotide repeats, due to their higher number of repeat units, had a greater contribution to the genome fraction occupied by SSRs: the cumulative sequence length of di- and tetranucleotide repeats was 570 kb and 432 kb, respectively.

### Distribution of SSR types in transcript sequences

We analyzed frequencies of perfect microsatellites in three transcript sequence datasets of cucumber which included two clustered EST datasets; an 18.8Mbp-set from cucumber inbred line WI 1983 flowers (Cucurbit Genomic Database, CuGenDB) and a 42.1 Mbp-set from Gy14 root and leaf tissues (http://cucumber.vcru.wisc.edu/; Weng et al., unpublished data); and a bulked EST dataset from WI 1983 (63.7 Mbp from CuGenDB). These data, along with similar analyses in clustered ESTs (gene indices) from seven reference plant species, are shown in Table 1. For comparisons with other species only the clustered ESTs dataset of cucumber was used, unless otherwise stated.

SSR frequencies in the two clustered and one non-clustered EST datasets of cucumber were comparable for tri-, hexa-, hepta- and octanucleotides, whereas a higher frequency of dinucleotides was found in clustered ESTs (16.3 vs 12.9 for WI 1983, Table 1). Comparable frequencies were also found for tetra- and pentanucleotides in both -clustered and bulk- WI 1983 datasets, whereas these repeats were less abundant in Gy14 ESTs. Biases due to variations in genotype, developmental stage and type of the tissue used for transcripts isolation, and sequencing technologies may account for the observed differences. Considering only EST datasets of WI 1983, which used the same genotype, transcripts assay protocol and sequencing technology, the overall SSR density was slightly higher in clustered (458 SSRs/Mbp) than in bulk (428 SSRs/Mbp) ESTs.

The overall microsatellite density in cucumber ESTs was lower than in genomic sequence, although not significantly (*P *= 0.41). Cucumber trinucleotides and hexanucleotides were more frequent in transcripts than in genomic DNA, regardless of the EST dataset used. Trinucleotides accounted for 42.6-48.5% and 44.6% of the total SSRs in clustered and bulk ESTs, respectively (Table 1). Tetra-, di-, penta-, hexa-, hepta- and octanucleotides followed, in that order, in abundance. Analysis of microsatellites in the expressed fraction of the genomes of other species revealed distributions similar to cucumber, with a rather constant relative abundance of repeat classes across taxa: trinucleotides were by far the most frequent SSR type (representing 42 to 66% of all SSRs), followed in order by tetra-, di-, hexa-, penta-, hepta-, and octanucleotides, in nearly all the species analyzed (Table 1). However, the absolute frequency of trinucleotides varied by more than a 4-fold magnitude across plant species, ranging from 108 in grapevine to 486 trimers per Mbp in rice. Trinucleotide density in cucumber was most similar to that of soybean, poplar and *Medicago*, the most closely related species included in the study. Considering all the species, di-, tetra-, penta-, and heptanucleotide repeats were significantly more abundant in genomic compared to EST data (*t*-test, *P *< 0.05). On the contrary, tri- and hexanucleotides were more frequent in ESTs than in genomic sequences, although these differences were not significant.

### Distribution of microsatellite motifs

We have carried out a detailed analysis of individual repeat motifs for each type of SSRs found in genomic and EST sequences of cucumber, along with similar calculations for other plant species, which is presented as supplemental data (Additional file 1) available with the online version of this paper. Here we present the main results of this analysis.

#### Dinucleotide motifs

**Figure 2:**
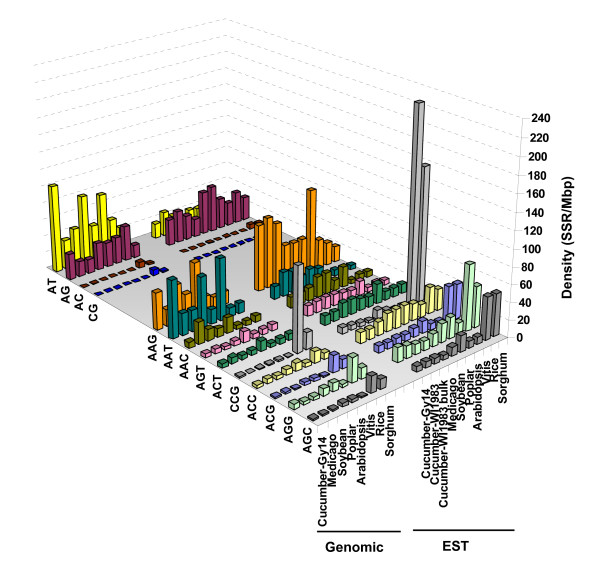
**Distribution of different di- and trinucleotide repeats in genomic and EST sequences of cucumber and selected seven other plant species**. Frequency values were expressed as number of repeats per million base pairs of sequence. Species were arranged in order to their phylogenetic relationship with cucumber. All species considered, repeats of AT (*T = *4.53, *P = *0.0021), AC (*T = *1.34, *P = *0.018), and AAT (*T = *2.68, *P = *0.028) were significantly more frequent in genomic - than in EST data, whereas ACT (*T = *4.25, *P *< 0.001) and ACC (*T *= 7.09, *P *< 0.001) were more abundant in EST sequence. Detailed information on frequencies of individual di- and trinucleotide repeat motifs was provided in Additional file 1 (supplement Tables S2a and S3a).

Analysis of AC, AG, AT and CG repeats showed that AT motif was dramatically overrepresented in cucumber genomic sequences (Figure 2 and Additional file 1, Table S2a). AT repeats were not only the predominant dinucleotides, but they were also the most frequent motif in the entire genome, accounting for 18.7% of the total SSRs. In addition, compared to other species, cucumber had the highest density of AT repeats, with AT also being the most abundant single motif, regardless of repeat type, across all genomic datasets examined. In contrast, cucumber EST sequences had a much lower frequency of AT dinucleotides, with AG being the predominant EST dinucleotide.

The same general pattern of dinucleotide motifs distribution was observed in other species; AG predominated in EST sequence whereas AT prevailed in genomic DNA (Figure 2). AC and CG repeats were the least frequent dinucleotides (in that order) in both genomic and EST sequences of all species. Detailed frequencies of individual dinucleotide motifs are included in Additional file 1 Tables S2a and S2b.

#### Trinucleotide motifs

In cucumber, as well as in other species analyzed, trinucleotide repeats were more abundant in ESTs compared to genomic sequences. Analysis of the frequencies of different trinucleotides revealed that repeats of AAT and AAG were most common in cucumber genomic data, whereas AAG predominated in ESTs (Figure 2). This pattern also applied for all dicots. Conversely, CCG were the rarest trinucleotides in both genomic and EST datasets of dicot species including cucumber. The opposite distribution was observed in the monocots rice and sorghum, for which CCG was by far the most abundant trinucleotide repeat motif in both genomic and EST data. A strong bias in the distribution of trinucleotides towards GC-rich motifs was found in genomic and protein-coding sequences of monocots, such bias being more evident in EST sequence (Figure 2). For example, GC-rich motifs accounted for on average 78% of the trinucleotide repeats in monocots, with a range of 56% (in sorghum genomic data) to 88% (in rice ESTs), whereas only 33% of GC-rich trimers were found in dicots, ranging from 10 (in grapevine genomic data) to 46% (in poplar ESTs). For details, see Additional file 1, Tables S2a, b and S3a, b.

#### Tetranucleotide motifs

**Figure 3:**
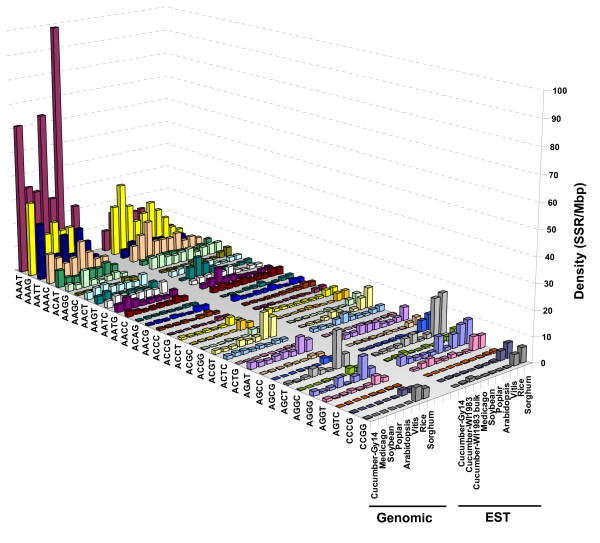
**Distribution of tetranucleotide repeats in genomic and EST sequences of cucumber and seven other plant species**. Frequency values are expressed as number of repeats per million base pairs of sequence. Species are arranged in order to their phylogenetic relationship with cucumber. All species considered, repeats of AAAT (*T = *3.46, *P = *0.009), AATT (*T = *3.90, *P = *0.005), AAAC (*T = *2.71, *P = *0.017), ACAT (*T = *3.07, *P *= 0.008), AACT (*T = *4.04, *P *= 0.001), AATC (*T = *3.67, *P *= 0.003), and AATG (*T = *2.25, *P *= 0.041) were significantly more frequent in genomic- than in EST sequences, whereas the AACC (*T = *2.39, *P *= 0.031) tetranucleotides were more abundant in EST data. Refer to Additional file 1 (supplement Tables S2a and S3a) for details on frequencies of individual tetranucleotide motifs.

Tetranucleotide repeats were far more frequent in genomic compared to EST sequences of cucumber (Table 1). The AT-rich motifs AAAT, AAAG, AATT, and AAAC were, in that order, the most abundant tetranucleotides in cucumber genomic data, together representing ~78% of all tetramer repeats, whereas GC-rich repeats like CCGG, CCCG, AGCC, and AGCG were the rarest, with relative frequencies of less than 0.1% (Figure 3, and Additional file 1, Table S2a). A similar distribution was observed in the other dicots, for which a clear predominance of these same AT-rich motifs was observed. In general, the same tetramer repeats that predominated in genomic sequences of dicots, including cucumber, also prevailed in their ESTs counterparts (Figure 3, and supplement Table S2a). As in the case of trinucleotides, monocot species had a much higher density of GC-rich tetramers than dicots in both genomic and EST sequences (see Additional file 1, Table S2a for details).

#### Pentanucleotide motifs

Cucumber genomic sequences had higher frequency of pentanucleotide repeats than ESTs. AAAAG, AAAAT, AAATT, AAAAC and AATAT were, in that order, the most common motifs in the cucumber genome, together accounting for > 65% of the total pentanucleotides (see Additional file 1, Table 2b). In general, these motifs also predominated in cucumber ESTs and, compared to other species, cucumber had the highest density of AAAAG repeats in both genomic and EST sequences.

Analysis of pentanucleotide frequencies in genomic data from other species revealed that, in all dicot species except cucumber, AAAAT was the most abundant repeat, outnumbering the next most frequent repeats, AAAAG or AAAAC, by 2.2 to 4 fold, respectively, whereas nearly identical frequencies for AAAAG and AAAAT were found in monocots. Similar to monocots, cucumber genomic sequences had a nearly 1:1 ratio for these two major pentanucleotide motifs, (although their absolute frequencies in cucumber were roughly 3-7 fold higher than monocots).

In general, DNA sequences of monocot species had a higher frequency of GC-rich pentanucleotide repeats compared to dicots, although such differences were more evident when comparing EST data sets. Repeats of AGAGG, AGGGG, AGGCG, AGAGC, CCGCG, and CCCCG were relatively abundant in rice and sorghum, but markedly underrepresented in dicots (AT-rich pentamers predominated in the latter) (see Additional file 1, Tables 2b and 3b).

#### Hexanucleotide motifs

Using our SSR search parameters, a slightly higher frequency of hexanucleotide repeats was found in cucumber ESTs (NCBI dataset) than in genomic sequence. AT-rich hexanucleotide motifs such as AAAAAG, AAAAAT, AAAAAC, and AAAATT predominated in cucumber genomic data (73% of total), whereas in ESTs hexanucleotide repeats were more evenly distributed between AT-rich motifs (28-37%) like AAAAAG, AAACAC and AAGATG; and GC-rich (~31%) and AT/GC balanced (32-42%) motifs such as AAGAGG, AAGGAG, and AGCCGG.

Cucumber genomic sequence had an overall density of hexanucleotide motifs similar to that of poplar, grapevine, rice and sorghum, and nearly twice the density found in *Medicago*, soybean and *Arabidopsis*. The AAAAAG repeat was at least two-fold more frequent in cucumber than in other species. As observed in cucumber, EST sequence data from all others species had a higher frequency of GC-rich and AT/GC balanced motifs as compared to their genomic counterparts (*T *= 2.58, *P *= 0.031), with rice and sorghum ESTs having the highest density, and *Medicago*, soybean and *Arabidopsis *the lowest density of such repeats (Additional file 1, Tables S2b and S3b).

#### Hepta- and octanucleotide motifs

Hepta- and octanucleotides were the most underrepresented repeat types in cucumber genomic and EST data. Heptanucleotides were approximately 3 times as dense as octanucleotides. Both repeat types were ~ 35% more frequent in genomic than in EST sequences of cucumber. Analysis of motifs revealed -for both SSR types- a rather dispersed frequency distribution in cucumber genomic sequence, as indicated by the presence of many different motifs at low frequency. AT-rich repeats of AAAAAAG, AAAAAAT, AAAAAAC, AAAATTT, and AAAGAAG were most abundant, together accounting for 30% of the total genomic heptanucleotides, whereas AAAAAAAG, AAAGAGAG, AAAAAAAT, AAAAGAAG, and AAAAAAAC motifs prevailed among octanucleotides. The same heptanucleotide motifs predominated in cucumber clustered ESTs, except for AAAATTT, whereas repeats of AAAAAAAG, AGAGAGGG, AAAAAAAC, and AAAGAGAG were most frequent among transcripts octanucleotides.

In other plant species, a similar relative abundance of hepta- and octanucleotide repeats was found in genomic sequence compared to EST data. Again, in general, many different motifs with low individual frequency were found in both genomic and EST sequence. Predominant heptanucleotide motifs in genomic sequences of most plant species were AAAAAAT, AAAAAAG, AAAAATT, AAACCCT, AAAATTT; whereas AAAAAAAT, AAAAAAAG, and AAAAAAAC were frequent among octanucleotides. In EST sequence, a modest prevalence of AAAAAAAT, AAAAAAAG, and AAAAAAAC motifs was found in dicots, whereas motifs of CCCGCCG, AGCGGCG, and ACTCATC were more common in rice and sorghum.

### In silico analysis of SSR polymorphism between Gy14 and 9930 cucumber inbred lines

Using the 2,099 SSR primer pairs originally designed from genomic sequence of the cucumber inbred line 9930 [38], 1,542 (73.5%) specific bands were amplified *in silico *from the genome sequence of Gy14. The remaining 557 markers failed to generate specific amplicons as they did not meet one or more of the criteria needed for "specific PCR products" (see Methods). On the basis of amplicon length comparison between 9930 and Gy14 products, the SSRs were classified as polymorphic (when both alleles differed in at least 2 bp) or monomorphic (both amplicons were of identical size). Because a fraction of the Gy14 sequences was generated from paired-end clones, and the precise sequence length between these 'ends' was uncertain, we were not able to make reliable comparisons of amplicon sizes in those cases where the primers of a particular SSR mapped to different ends of the paired-end contig. This subset included 407 SSRs that could not be assigned to either monomorphic or polymorphic categories. In addition, the Gy14 amplicons of 129 SSRs varied from its corresponding 9930 amplicons by only 1 bp. Due to the possibility that PCR or sequencing errors may be responsible for some of these variations, these SSRs were removed from the analysis. The remaining 1,006 SSR loci, which included 972 simple SSRs and 34 compound SSRs, could be unambiguously evaluated for size polymorphisms comparing Gy14 and 9930. Information on SSR marker names, primer sequence, *in silico *PCR results from Gy14 and 9930 genomes of these 1,006 SSRs was provided in Additional file 1, Table S4.

Of the 1,006 SSR loci, 520 (51.7%) were monomorphic and 486 (48.3%) were polymorphic, as indicated by *in silico *comparisons between Gy14 and 9930. The two SSR data sets, monomorphic and polymorphic, were further analyzed to investigate a possible relationship between polymorphism level and repeat length, for each SSR type (Table 2). The fact that only 981 of the 1,006 SSR loci are included in Table 2, is because heterogeneous compound SSRs (25 SSRs) could not be assigned to a repeat-type class. Regardless of the basic motif length and sequence, the monomorphic SSRs had a higher frequency of short repeats (30.6% vs. 18.0%, for repeats < 30 nucleotides (nt)) whereas the polymorphic SSRs included a substantially higher percentage of long repeats (33.6 vs 20.8%, for repeats > 40 nt). Both groups had similar content of intermediate-size (30-40 nt) repeats (monomorphic 48.8% vs. polymorphic 48.4%, Table 2).

**Table 2:** Frequency distribution of cucumber microsatellites, by repeat length, in monomorphic and polymorphic SSR data sets.

		Repeat length				
				
SSR data set	Repeat type*	< 30 nt	30-40 nt	> 40 nt	Total	%
**Monomorphic**	**Di-**	34	29	13	76	**14.8**
	**Tri-**	95	119	63	277	**53.9**
	**Tetra-**	26	38	10	74	**14.4**
	**Penta-**	0	33	10	43	**8.4**
	**Hexa-**	1	32	11	44	**8.6**
		
	**Total SSRs (%)**	156 **(30.6)**	251 **(48.8)**	107 **(20.8)**	**514 (100)**	
**Polymorphic**	**Di-**	42	102	75	219	**46.9**
	**Tri-**	21	67	49	137	**29.3**
	**Tetra-**	21	24	13	58	**12.4**
	**Penta-**	0	18	3	21	**4.5**
	**Hexa-**	0	15	17	32	**6.9**
	**Total SSRs (%)**	84 **(18.0)**	226 **(48.4)**	157 **(33.6)**	**467 (100)**	

* No hepta- or octanucleotide repeats were present in the SSRs sample used in this analysis. Loci with compound SSRs were excluded from the analysis unless all simple repeats within the compound SSR were of the same type, in which case 'repeat length' was the sum of the repeat lengths in each simple repeat (for example, (AT)_8_(GT)_12 _= 40 nt). Mononucleotides (5 SSRs) were not included in the analysis.

Considering the basic repeat types, dinucleotides (47%) were the most common polymorphic SSRs with a frequency 3-fold higher than the frequency of dinucleotides found in the monomorphic group (Table 2). On the other hand, trinucleotides (53.9%) predominated among monomorphic microsatellites, and tri- and pentanucleotides were almost twice as frequent in this group as compared to the polymorphic SSRs. Variation in the distribution of repeat motifs associated with monomorphic and polymorphic microsatellites was also found. AAG/CTT (30.7%), AAT/ATT (14.7%), and AAAG/CTTT (9.1%) were the most frequent motifs among monomorphic SSRs, whereas AT/AT (36.1%), AAG/CTT (16.8%), and AG/CT (9.3%) predominated among polymorphic SSRs (data not shown). The nucleotide composition of repeats in both groups was similar, with 83.4% and 83.8% of AT-rich repeats in the monomorphic and polymorphic datasets, respectively.

**Figure 4:**
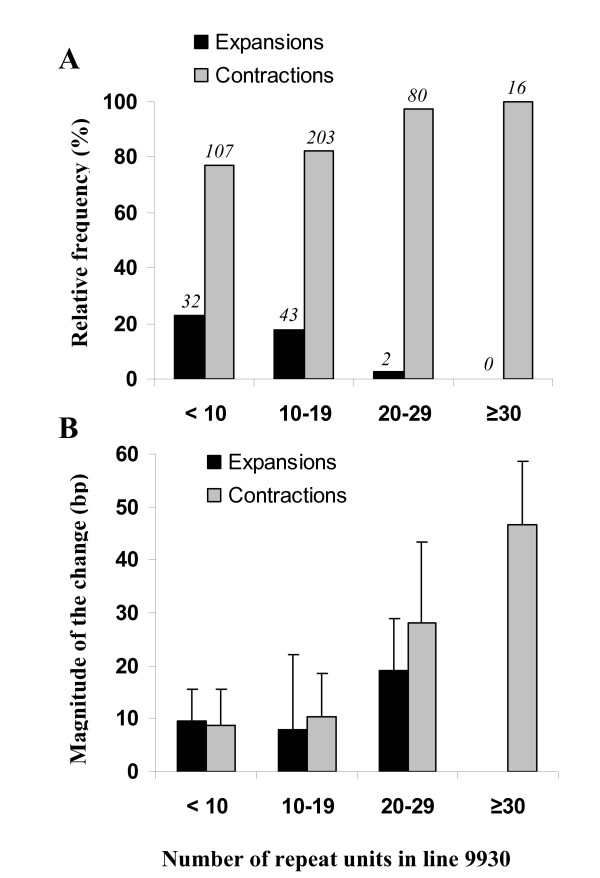
**Frequency (A) and magnitude (B) of SSR expansions/contractions in cucumber genotypes Gy14 versus 9930 in relation to the number of repeat units of the microsatellite**. The allele from cucumber inbred line 9930 was considered as the reference (or initial state) of the repeat. For each class of 'number of repeat units', the percentage (bars) and actual counts (italic numbers) of SSRs showing expansions or contractions in Gy14 are presented in panel A. The magnitude of the change in allele sizes (∆*al*) from 9930 and Gy14 is presented in panel B (error bars denote standard deviations). For compound SSRs, repeat units in each uninterrupted repeat were summed if the allele had at least 10 repeat units (e.g., (AT)_8_(GT)_12 _= 20 repeat units).

**Figure 5:**
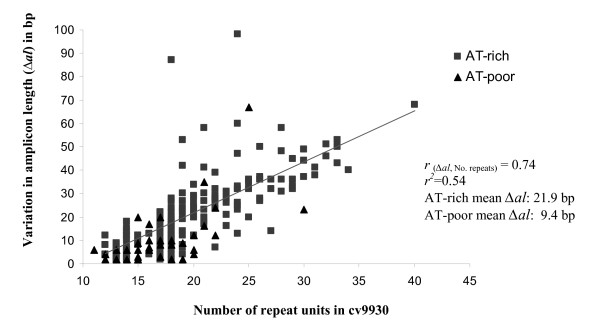
**Relationship between amplicon length differences (∆*al*) between Gy14 and 9930, and the number of repeat units in dinucleotides repeats**. AT-poor SSRs include AT:GC balanced repeats and GC-rich motifs. Loci with compound SSRs containing repeats other than dinucleotides were not considered. Loci with compound SSRs were excluded from the analysis unless all simple repeats within the compound SSR were dinucleotides, in which case the number of repeat units was the sum of the repeat units in each simple dinucleotide, for example, (AT)_8_(GT)_12 _= 20 repeat units.

Further analysis of the polymorphic SSR dataset was performed. We observed that most of the SSR alleles from 9930 had a corresponding Gy14 allele that was shorter (*i.e.*, a "contraction" if the 9930 allele is arbitrarily considered the 'initial' state of the repeat), and that the relative frequency of contractions and expansions varied in relation to the repeat length of the SSRs, with an increasing proportion of contractions found for longer repeats (Figure 4). The magnitude of the change in allele length (∆*al*) was also related, positively and significantly (*r *= 0.62, *P *= 0.005), to repeat length. The latter general trend was largely due to the contribution of dinucleotides which showed a moderately strong, positive, and significant relationship (*r *= 0.74, *P *< 0.0001) between ∆*al *and repeat length (Figure 5). This association was not statistically significant for other repeat types. Dinucleotides also had a statistically higher mean ∆*al *(19.2 bp, 95% LSD test) than tri- (8.5 bp), tetra- (8.7 bp), penta- (8.4 bp), and hexanucleotides (12.3 bp), with the latter repeat classes not being significantly different from each other (data not shown). Because variation in microsatellite mutation rates and polymorphism has been previously associated with the nucleotide composition of the repeats [45], we compared the ∆*al *of AT-rich and AT-poor repeats for each SSR type. The mean ∆*al *for AT-rich dinucleotides was significantly higher than for AT-poor dinucleotides (21.9 bp versus 9.4 bp, 95% LSD test) (Figure 5), whereas no significant variation was found for ∆*al *between AT-rich and AT-poor repeats in other SSR types.

### Experimental validation of in silico PCR results

As indicated by results from our *in silico *analysis, most alleles from the cucumber inbred line 9930 were larger than the corresponding alleles from Gy14. These findings were experimentally validated in a subset of these markers. A total of 660 SSR primer pairs evaluated *in silico*, which included 317 polymorphic and 343 monomorphic markers, were tested for PCR amplifications using Gy14 and 9930 genomic DNAs as templates, and the PCR products were resolved with 6% polyacrylamide gel electrophoresis (PAGE). Overall, agreement between *in silico *and empirical microsatellite data was found for 640 (97%) markers. The remaining 20 SSRs (3%) revealed disagreements between both types of analyzes. Of these, 11 SSRs were polymorphic *in silico *and monomorphic in PCR analysis in the lab, whereas 9 markers were monomorphic *in silico *and polymorphic by wet-lab PCR assays. Among the evaluated SSRs that were polymorphic *in silico *and empirically (306 markers), 191 (62.4%) SSRs amplified shorter amplicons in Gy14 than in 9930, and 115 (37.6%) showed the opposite, which was fully consistent with the results from our analysis *in silico*.

### Primers design for new SSR markers

Primer pairs for 83,472 microsatellite loci were designed from cucumber (Gy14) genomic sequence (additional files 2 and 3, supplement **Table S5**). Primer pairs that generated more than one *in silico *PCR product were discarded. Thus, this large set of putative markers includes only non-redundant (single copy) microsatellites. The exact positions of these SSRs in the Gy14 genome assembly, as well as information on repeat motifs, expected PCR product length, and primers *Tm*, are presented in Table S5. In addition, correspondences with SSR markers previously developed and mapped by Ren et al. (2009) [38], and their linkage map positions, if available, were included in Table S5 as well.

## Discussion

### Frequency and distribution of SSRs in the cucumber genome

Genome-wide analysis of simple sequence repeats, coupled with information on their distribution in coding and non-coding regions may provide insights into possible roles of SSRs in gene regulation and genome organization, and provide abundant markers for genetic, genomic and evolutionary studies. In this study, we analyzed the distribution and frequency of perfect microsatellites with motifs of 2-8 bp long and minimum of 3 repeat units in the assembled cucumber Gy14 genomic DNA sequences representing 55% of its genome, as well as in datasets of EST sequences. It was found that microsatellites contributed significantly to the assembled cucumber genome (~0.9% of the 367 Mbp cucumber genome). A general negative correlation between genome size and SSR density in plants has been reported [19]. Our data in cucumber agrees with this general trend. Having a genome size roughly comparable to that of rice (370-490 Mbp) [46], poplar (485 Mbp) [47], and grapevine (487 Mbp) [48], cucumber (367 Mbp) [44] had an SSR density similar to these species, whereas they all had a higher repeat density compared to the larger genome of sorghum (818 Mbp) [49] and soybean 1,115 Mbp) [44], such difference being ~4 fold higher when compared to the large 2,365 Mbp genome of maize (120 SSRs/Mbp), and the 1,000 Mbp chromosome 3B of wheat (163 SSRs/Mbp) [50]. *Arabidopsis thaliana *deviates from this general trend, since its 157 Mbp genome, the smallest in our dataset, harbors an SSR density (370.5 SSRs/Mbp) substantially smaller than densities found in plant species with twice (e.g., rice) and three times (e.g., poplar, grapevine) larger genomes.

Differences in SSR density between genomic and the EST sequences of cucumber and other species were found (Table 1). In cucumber genomic DNA, microsatellites are present at a higher density than in coding regions (1.2-1.5 and ~ 1.3 fold higher compared to clustered and bulk ESTs, respectively). Among all the species, cucumber, poplar, and grapevine had a 1.2 to 2.0-fold higher repeat density in genomic compared to EST sequence, whereas the opposite was found for soybean, rice and sorghum, which had 1.2 to 2.0-fold higher density in ESTs than in genomic data. SSR density was nearly equal in both sequence datasets for *Medicago *and *Arabidopsis *(Table 1). These results partially contradict those of Morgante et al. (2002) [19] reporting a higher microsatellite frequency in transcribed sequences than in genomic DNA of five plant species. Conversely, Mun et al. (2006) [51] reported that four of the five plant species examined by them had 1.3 to 2.8-fold higher SSR frequency in genomic than in bulk EST sequences. More broadly, Toth et al. (2000) [3] examined SSR frequencies in several eukaryotic organisms ranging from yeast to primates including *Arabidopsis thaliana *as a plant representative, and reported higher overall SSR density in intergenic regions and introns as compared to exons in all the taxa investigated. Variations inherent to the genomes examined in these studies may account for large part of the observed differences. However, some of these differences may also be due to variations in the search parameters and algorithms used for detection of microsatellites in DNA sequence, as well as differences in the type and size of the data sets used. For example, the higher repeat density found in ESTs compared to genomic sequence by Morgante et al. (2002) [19] was suggested to be due to a bias in the estimation of frequencies due to the small data set used [51]. However, additional factors are more likely to contribute to the different results obtained by Morgante et al. (2002) [19] and Mun et al. (2006) [51]. For example, different repeat types (mono- to pentanucleotides *versus *mono- to octanucleotides) of different minimum lengths (12 bp and 3 repeat units, *versus *only 12 bp) were searched using different software. Significant variation has been found among algorithms used for SSR detection [52]. Toth et al. (2000) [3] used custom Perl scripts to detect mono- to hexanucleotide SSRs of at least 12 bp and two repeat units, and expressed the results in bp of repeats/Mbp of sequence (instead of count/Mbp). These seemingly minor differences in procedure can strongly influence microsatellite distributions and impede direct comparison among studies. For example, in the present study, the density of hexanucleotides ranged from 13 to 56 per Mbp across genomic and EST sequences of all species (representing 4-12% of the total SSRs), whereas data from Mun et al. (2006) [51] showed that hexanucleotides < 19 bp long were the most frequent repeat type in genomic and EST data, with densities ranging from 597 to 1,015 hexamers/Mbp, representing a 2.1 to 6.9-fold increase in repeat density compared to mono-, di-, tri-, tetra- and pentanucleotides. Such extraordinary differences are mainly due to the fact that we considered a minimum of 12 bp and 3 repeat units per SSR, whereas 12 bp (regardless of the number of repeat units) was the cutoff for minimum SSR length in Mun et al. (2006) [51]. Therefore, while we discarded hexamers of 2 repeats (12 bp), they were kept in analyses by Mun et al. (2006) [51]. Because the majority of hexanucleotides occur in the form of two repeated units, these SSRs outweighed other types of repeats, increasing also the overall SSR density in their study. This was confirmed by the fact that when we repeated the analysis using 2 repeat units (instead of 3) as the minimum length, we obtained similar values as reported by Mun et al. (2006) [51]. For example, hexanucleotide density was 845, 727, and 801 repeats/Mbp for cucumber, *Arabidopsis *and rice, respectively (data not shown). The fact that abundant hexanucleotides with only two repeat units occur in most genomes is also reflected in the data presented by Toth et al. (2000) [3]. Although hexamers with 2 repeat units = 12 bp were also included in their analysis, because SSR frequencies were expressed as 'total SSR length/Mbp' (instead of number of SSRs/Mbp), the mostly-short hexamer repeats only accounted for ~ 10% (mean from all taxonomic groups; with 6.6% for plants) of the total SSR content. This result by Toth et al. (2000) [3] is in good agreement with calculations for hexanucleotide frequencies relative to other repeat types from the present study. Our criterion for selecting a minimum of three repeat units per SSR was based on the fact that polymorphism levels and mutation rate correlate positively with the number of repeat units [53], and therefore, more useful polymorphic markers are expected to be developed from these SSR loci. The above considerations point to the importance of critically examining microsatellite frequency data, especially when such data was generated by different research groups and is intended for comparison purposes.

Trinucleotide repeats prevailed in protein-coding sequences of cucumber and all other species. An overrepresentation of trinucleotides in protein-coding sequences has been previously reported for several plants species [19,51,54-56], as well as in a range of other eukaryotes including humans and insects [3,18,57]. Although together with trinucleotides some reports also indicated a predominance of hexanucleotides in protein-coding sequences [3,18,51], this has not been consistently reported due to differences in the program parameters and criteria used for microsatellite detection, as discussed above. The relative abundance of trinucleotides over other SSR types has been attributed to negative selection against frame-shift mutations in the coding regions and positive selection for specific single amino-acid stretches [19].

As shown in Figures 2 and 3, within a given type of SSR there may be striking differences in the frequency of individual repeat motifs, within and between sequence datasets (genomic and EST) of a species. In cucumber, the base composition of the SSR motifs is strongly biased towards A's and T's in both EST and genomic data. For example, the density of the dinucleotide AT was > 1,400-fold higher than CG in genomic sequence (CG dimers were absent in bulk EST data), whereas AT dimers were also much more frequent (3.5 fold) in genomic compared to EST data. Similarly, in the case of trinucleotide repeats more than a 40-fold difference in density between the most frequent (AAT) and the least frequent (CCG) motifs was found in cucumber genomic sequence, whereas such difference was > 1,200-fold between the most (AAAT) and least (CCGG) frequent tetranucleotide motifs (Additional file 1, Table S2).

AT-rich repeats prevail in dicot species, including cucumber, but not in monocots. These differences may be explained, at least partially, by the relative nucleotide composition of their genomes (the average GC content in genomic sequence of dicots and monocots is 34.6% and 43.7%, respectively). However, these differences cannot explain, *per se*, the dramatic variation in the frequency observed for some particular motifs between both groups of plants. Most noticeably, genomic sequence of rice and sorghum had, on average, a 56-fold higher density of CCG trinucleotides compared to the average density for this repeat in dicots. Other motifs common in monocots but relatively rare in dicots were ACG, AGG, AGC, AGCT, AGGT, CCCG, CCGG, ACGT, and CCGCG. Whether these differences in base composition of microsatellite motifs played any role during specialization of monocots and dicots, is unknown.

### SSR polymorphism between Gy14 and 9930 cucumbers

Although both slippage of the DNA polymerase and unequal crossing-over have been proposed as possible mechanisms for microsatellite evolution, the first mechanism, which generates gains or losses of one or a few repeat unit(s), accounts for most of the mutations in microsatellites [58,59]. Our *in silico *pair-wise comparisons between 9930 and Gy14-derived alleles at 1,006 SSR loci allowed us to gain insights into possible relationships between the degree of polymorphism and particular features of cucumber microsatellites. We observed that longer alleles of 9930 (i.e., alleles with larger number of repeat units) showed a higher frequency of contractions in the corresponding Gy14 alleles, than short alleles of 9930. Despite the arbitrary assignment of an 'initial' state of the SSR, these results suggest that long repeats are more prone to decrease in size than are shorter ones. A similar trend was found in human [60] and yeast [61] microsatellites, with long alleles tending to mutate to shorter lengths more often than short alleles. Assuming that within-locus variations in repeat length are mainly due to mutations representing small changes in repeat number [62,63], large differences in allele sizes (∆*al*) from 9930 and Gy14 suggest that these loci have higher mutation rates and are more polymorphic than loci with small ∆*al *values. Indeed, the significant correlation found between the number of repeat units in the SSR and ∆*al *suggests a positive relationship between mutation rate, polymorphism and repeat length for these cucumber SSRs. This result is consistent with earlier studies showing positive and significant relationships between repeat length and mutation rate in human [53], fruit fly [64], and yeast [61] microsatellites. However, the strength of this association varies depending on the type of repeats. Data from human, mouse, fruit fly, and yeast microsatellites indicate that polymerase slippage rates are highest in dinucleotides, followed by tri-, and tetranucleotides [61,65]. Concordantly with these data, in the present study, the strongest relationship between ∆*al *and repeat length in cucumber was found in dinucleotides, and the frequency of dinucleotide repeats was 3 times higher among polymorphic SSRs than among monomorphic SSRs (Table 2). In addition, dinucleotide AT-rich repeats were more polymorphic than AT-poor dinucleotides, as indicated by their ∆*al *mean values and their relative frequencies in the polymorphic SSR dataset (36.2% for AT-rich versus 9.7% for AT-poor dinucleotides, data not shown), although such differences were not found in other cucumber SSRs. According to Schlötterer and Tautz (1992) [45] sequences with a high AT content are particularly unstable.

In this study, most SSR alleles from 9930 showed length contractions in the corresponding Gy14 alleles (the mean number of repeat units/SSR was 15.4 and 5.3 for 9930 and Gy14, respectively). We speculate that this is due to a bias in the selection of the SSR loci analyzed, rather than because of differences in SSR mutation rates between the two genotypes. Because, typically, during SSR marker development, there is reiterative selection for larger repeats. That is, library screening methods for SSR isolation are designed to yield a higher proportion of long SSRs. Once SSR sequences are obtained, the larger repeat tracks are -preferentially- used for designing primers, which are then pre-screened for their polymorphism. Thus, the most polymorphic loci are selected for further utilization (because there is a positive relationship between SSR length and polymorphism [53,61], there is selection for large repeat tracks at this stage too). The markers developed by Ren et al [38] represent a biased selection towards those SSRs with the most repeat motif numbers in 9930 and, therefore, are likely to harbor shorter repeats in Gy14. Indeed, these SSRs were originally selected -from genomic DNA sequence of 9930- on the basis of their longest motifs, and had an average repeat length of 39.4 bp [38]. Following this argument, the inverse scenario -i.e., SSR markers developed from the longest repeats of Gy14- should reveal shorter alleles in 9930, compared to Gy14. To test this hypothesis we mined the Gy14 genome sequence for microsatellites with motif lengths > 30 bp, and searched for the corresponding SSR loci in the 9930 genome sequence, and -for each locus- the motifs from Gy14 and 9930 were compared. A total of 323 Gy14 SSRs, with correspondences in the 9930 genome, were found, of which 316 SSRs (97.8%) had longer alleles in Gy14 compared to 9930, 1 SSR (0.3%) was longer in 9930, and 6 SSRs (1.9%) had same allele length in both genomes (data not presented). These results strongly support our previous hypothesis regarding an ascertainment bias -rather than differential SSR mutation rate- as the cause for the differences in repeat lengths found in 9930 and Gy14. The effects of such biases, have been well illustrated in several eukaryotes by Ellegren et al. [66,67] and Fitzsimmons et al [68], demonstrating that repeat lengths tend to be longer in the species from which markers were developed.

A total of 660 SSRs evaluated *in silico *were validated by PCR and gel electrophoresis. The experimental results were largely consistent with the *in silico *PCR data. However, a small fraction (20 of 660, 3%) of the markers showed different allele size and/or number of alleles compared to the corresponding *in silico *results. Potential sources of errors that may account for these discrepancies include PCR artifacts (e.g., non-specific amplifications, preferential amplification of one allele over another, etc.), post amplification gel-electrophoresis artifacts (e.g., secondary conformations and heteroduplexes, etc.), or errors in the estimation of amplicons size by us (this work) or by Ren et al. [38]. Errors in sequencing and/or assembly of the 9930 genome also seem possible. Indeed, we sequenced the Gy14 and 9930 PCR products from those SSRs that were polymorphic *in silico *but empirically monomorphic, and found that the repeat numbers of the SSR motifs in 9930 genome were not the same as shown in the draft genome (data not shown).

Consistent with results from our *in silico *analysis, the microsatellite experimental data revealed larger alleles in 9930 compared to Gy14 for most SSRs tested (62.4% of all polymorphic SSRs), thus providing empirical support for our hypothesis regarding a bias due to a prior selection of larger microsatellite loci in 9930.

### Utilization of new SSR resources for cucumber research

The high frequency of microsatellites found in cucumber genomic sequence compared to other plant species examined (Table 1) was unexpected. Due to the nature of the algorithms used for assembling sequence reads into contigs and scaffolds, the Gy14 assembly used in our analysis most likely corresponds to the single/low copy fraction of the genome (the left-aside unassembled sequence reads, presumably, represent most of the repetitive fraction). Thus, a possible explanation for the relative abundance of microsatellites in cucumber may rely on the fact that we analyzed the single/low copy portion of the cucumber genome. Given the nonrandom distribution of SSRs in plant genomes, predominating in non-repetitive sequences [19,54], it is possible that our data may represent an overestimate of the overall SSR density in the cucumber genome, since it was generated mainly from non-redundant genomic sequences.

The fact that ~48.3% of the SSR markers tested *in silico *were polymorphic between Gy14 and 9930 was also unexpected, considering that previous studies indicate 3-20% polymorphism at the DNA level within *Cucumis sativus *[25-27,41,42]. Most likely, this reflects the same situation discussed previously: markers selected for being highly polymorphic in 9930 will produce amplicons of different and shorter size in Gy14. Although these markers may be useful for fingerprinting and mapping, their use for estimating levels of genetic diversity in cucumber germplasm collections would provide an overestimate of the overall genetic diversity. To avoid this ascertainment bias and be able to achieve a representative picture of the genome-wide variation among germplasm, one should compare markers from both genotypes without previous selection. The recently finished genome sequences of 9930 and Gy14 provide an unprecedented opportunity for this type of analysis. Thus, genome-wide comparisons of simple sequence repeats or SNPs between 9930 and Gy14, may provide an unbiased picture of the overall DNA polymorphism in cucumber.

Due to the scarcity of highly polymorphic, user-friendly molecular markers in cucumber and other cucurbit crops, high-density genetic maps were not available until very recently [38]. Thus, most linkage maps with important trait loci have been developed with primarily low-throughput, anonymous, dominant markers such as RFLPs, RAPDs or AFLPs [28-31], making them difficult in applications like map-based cloning, and maker-assisted selection. Indeed, to date, only one economically important gene has been cloned in cucumber through the map-based cloning strategy (e.g., [69]), suggesting that marker assisted breeding in this species is still underdeveloped compared to some other horticultural crops (e.g., tomato). In the present study, we designed primer pairs for more than 80,000 non-redundant (single copy) cucumber microsatellites, and provide their exact positions in the Gy14 genome assembly. In addition, correspondences between these markers and those developed by Ren et al (2009) [38] were indicated (Table S5 in Additional files 2 and 3). This very large set of potential microsatellite markers, distributed throughout the genome, will facilitate the development of high-resolution maps for positional gene-cloning and detailed comparative mapping, among other applications.

## Conclusions

The current work has contributed to a detailed characterization of microsatellites in cucumber and their comparison to that of model species. The cucumber genome was found to be relatively rich in microsatellites, and polymorphism in comparing two cucumber inbreds was unexpectedly high, despite its distinctness as a species with low genetic diversity. Tetranucleotide repeats were the most frequent SSRs in the genome, with the dinucleotide 'AT' being the most common single motif. ESTs of cucumber were less frequent in microsatellites compared to genomic sequence, with trinucleotides strongly predominating in the formers. AT-rich motifs prevailed in both genomic and EST data. Using an electronic PCR strategy, we compared SSR amplicons from two cucumber genotypes. A surprisingly high percentage of polymorphic markers was found (~48%), with dinucleotides being the most polymorphic repeats. Our *in silico *analysis revealed a positive relationship between SSR polymorphism and the number of repeat units, a trend previously reported in other organisms. In addition, the data presented here increase the genomic resources available in cucumber by adding a large set (83,472) of microsatellite primers designed from microsatellite loci that are precisely located in the Gy14 assembly. These markers may facilitate a number of genetic and genomic studies in *Cucumis*, including anchorage of the sequence scaffolds to the genetic map, development of high-resolution linkage maps, positional gene-cloning, and comparative mapping in other cucurbit crops.

## Methods

### Source of genomic and transcript sequences

Gy14 is a gynoecious, inbred, classical North American pickling cucumber line with multiple disease resistance and superior horticultural characteristics. The genome sequencing of Gy14 was performed with the Roche 454 GS FLX Titanium platform at 36× genome coverage with combined shotgun (24×) and multi-span paired end (3-kb at 9× and 20-kb at 3×) *de novo *sequencing strategies. Using the Newbler assembler software version 2.3 (454 Life Sciences), high quality sequence reads were assembled into 4,219 scaffolds ranging in size from 500 bases to 6.9 Mbp with an average of 48,129 bases per scaffold. The assembled sequences, which totaled 203,052,159 bases, were used in this study to characterize the distribution of microsatellites in the cucumber genome. The completeness of the 203 Mbp assembly is supported by the mapping of over 99% of ~2 million EST reads (generated using 454-XLR technology) from Gy14 leaf and root tissues. The assembled Gy14 genome (4,219 scaffolds) and transcriptome are publicly accessible through http://cucumber.vcru.wisc.edu/ (BLAST searchable). The annotated Gy14 genome is accessible through the US Department of Energy's Joint Genome Institute (USDOE JGI) database at http://www.phytozome.net/cucumber.php#A.

For comparison purposes, the complete genome sequence of *Arabidopsis thaliana *L., rice (*Oryza sativa *L.), grapevine (*Vitis vinifera *L.), and poplar (*Populus trichocarpa *L.) were downloaded from the National Center for Biotechnology (NCBI) database (GSS Section). The recently finished sorghum (*Sorghum bicolor *L.) genome sequence was downloaded from the USDOE-JGI database. In addition, 243 and 110 Mbp of genomic sequences from soybean (*Glycine max *L.) and *Medicago truncatula*, respectively, were also downloaded from NCBI-GSS. Soybean and *Medicago *were included in the study because they represent more closely related taxa to cucumber, as they belong to the Fabaceae family, order Fabales, the latter being a sister clade to the Cucurbitales, within the Eurosids I [70].

As a source of transcript sequences we used plant gene indices of The Institute of Genome Research (TIGR), which are up-to-date non-redundant (unigenes) EST collections [71]. Thus, gene indices of *Arabidopsis *(74.8 Mbp), poplar (67.6 Mbp), grapevine (81.4 Mbp), *Medicago *(51.9 Mbp), soybean (51.3 Mbp), rice (158.2 Mbp) and sorghum (32.4 Mbp) were downloaded from the Gene Index databases (http://compbio.dfci.harvard.edu/tgi/plant.html) and searched for SSRs. Gene indices for cucumber were not available. Instead, we used 3 sets of cucumber ESTs for mining microsatellites including two clustered EST datasets; an 18.8Mbp-set from flower organ of cucumber inbred line WI 1983 [72] (Cucurbit Genomic Database, CuGenDB, http://www.icugi.org/) and a 42.1 Mbp-set from Gy14 root and leaf tissues (accessible from http://cucumber.vcru.wisc.edu/); and a bulk EST dataset from WI 1983 (63.7 Mbp; CuGenDB). Detailed information about the databases used for the present study is provided in Additional file 1, Table S1. The *t*-test statistic was used to compare SSR frequencies in genomic and EST data using the program STATGRAPHICS Centurion XV.

### Detection of SSRs and primer design

Although SSRs are commonly defined as repeats of 1- to 6-bp motifs [1], in this work we also analyzed repeats with 7- and 8-bp motifs, whereas mononucleotides were not considered due to the difficulty of distinguishing bona fide microsatellites from sequencing or assembly error (these biases are more common in whole genome shotgun sequence data), and because (A/T)*n *repeats in EST sequences may be confused with polyadenylation tracks. Thus, DNA sequences were searched for perfect microsatellites, with a basic motif of 2-8 bp, using the computer program MISA (MIcroSAtellite identification tool) [73]. Repeats with a minimum length of 12 (for di- to tetranucleotides), 15 (for pentanucleotides), 18 (for hexanucleotides), 21 (for heptanucleotides), and 24 bp (for octanucleotides) were recorded.

The positions of the SSRs found in the Gy14 genome assembly (scaffolds) were recorded, and oligonucleotide primers were designed for the genomic sequence flanking these SSRs using Primer3 (v. 1.1.4) software [74]. Primers were designed to generate amplicons of 100-400 bp in length (optimum 250 bp) with the following minimum, maximum and optimum values for Primer3 parameters: primer length (bp): 18-25-30; *Tm *(°C): 57-60-63; GC content (%): 30-40-60. Other parameters used the program default values.

### In silico analysis of SSR polymorphisms and experimental verification

The draft genome of the Chinese fresh market cucumber inbred line 9930 was recently released [37] (available at http://cucumber.genomics.org.cn/). From the 3× coverage Sanger shotgun sequence assembly of the 9930 genome, 2,112 microsatellite primer pairs with the longest repeats were designed and 966 of them were mapped [38]. In the present study, we used these SSR markers, with the exception of mononucleotides (13 SSRs), to investigate polymorphism level at microsatellite loci of cucumber. Using an *in silico *PCR (virtual PCR) strategy, 2,099 pairs of primer sequences from 9930 were mapped onto the Gy14 sequence scaffolds and the genomic sequence delimited by each primer pair (i.e., by the forward and reverse primers of each SSR) was extracted, analyzed, and annotated for the presence and type of microsatellite repeat and its sequence length. This was performed with a custom Perl script that used the NCBI BLASTN program as a search engine with expect value of 10 and no filtering. We allowed up to 5 nucleotide mismatches at the 5' end of the primer but no mismatches at the 3' end, and a minimum of 80% overall match homology. The program extracted products between 100 and 2000 bp. For a given primer pair, we considered that a specific amplicon was generated if both forward and reverse primers were mapped to the same scaffold, within a region of 4 kb, and they flanked an SSR with the same basic motif as the expected one in 9930. The specific *in silico-*generated amplicons from Gy14 were compared with the expected amplicon size from 9930 according to Ren et al. (2009) [38], and their size differences were recorded. On the basis of these size differences, the SSRs were classified as polymorphic if amplicons from Gy14 and 9930 varied in at least 2 bp, or monomorphic if both amplicons were of identical size. SSR loci with 1-bp difference were considered ambiguous and were removed from the analysis. The polymorphic and monomorphic SSR datasets were analyzed in more detail with MISA.

To experimentally validate the *in silico *PCR results, we synthesized primers for 660 SSRs (317 polymorphic and 343 monomorphic based on *in silico *results) and performed PCR amplification with genomic DNAs from 9930 and Gy14 as the templates. Seeds of cucumber inbred line 9930 were kindly provided by Prof. Xing-Fang Gu (Institute of Vegetables and Flowers, Chinese Academy of Agricultural Sciences, Beijing, China). For PCR amplification, genomic DNAs of Gy14 and 9930 were extracted from newly expanded young leaves using the CTAB method [75]. Each reaction contained 20 ng template DNA, 0.5 μM each of two primers, and 1× PCR master mix (Fermentas, Glen Burnie, MD, USA) in a total volume of 10.0 μl. A single, touchdown PCR program [76] was employed. The PCR products were resolved with 6% polyacrylamide gel electrophoresis (PAGE) and visualized with silver staining.

## Abbreviations

AFLP: amplified fragment length polymorphism; EST: expressed sequence tag; Mbp: million base pairs; PCR: polymerase chain reaction; SNP: single nucleotide polymorphism; RAPD: random amplified polymorphic DNA; SSR: simple sequence repeats.

## Authors' contributions

PFC performed the research, analyzed the data and wrote the manuscript. DAS designed and developed scripts for bioinformatic analysis, performed data analysis and participated in preparation and improvement of the manuscript. TTH and CDK leading teams sequenced the Gy14 genome and transcriptome. PWS participated in experimental design, coordinated the experiments, and improved the manuscript. SH provided SSR primer information from 9930 draft genome. LY conducted empirical validation of SSR polymorphism. YW conceived the study, designed the experiments, performed part of the PCR experimental verification, wrote and revised the manuscript. All authors read and approved the final manuscript.

## Supplementary Material

Additional file 1 **This MS Excel file includes supplemental Tables S1 to S4**. Supplement Table S1. Characteristics of genomic and EST sequence databases used. Supplement Table S2a. Distribution of di-, tri-, and tetranucleotide repeats in genomic and EST sequences of cucumber. Supplement Table S2b. Distribution of the most frequent penta-, hexa-, hepta- and octanucleotide repeats in genomic and EST sequences of cucumber. Supplement Table S3a. Distribution of di-, tri-, and tetranucleotide repeats in genomic and EST sequences of reference plant species. Supplement Table S3b. Distribution of the most frequent penta-, hexa-, hepta- and octanucleotide repeats in genomic and EST sequences of reference species. Supplement S4. Information of 1,006 SSRs used *in silico *PCR analysis Click here for file

Additional file 2 Supplement Table S5a: list of primers for 83,472 microsatellites found in *Cucumis sativus *Gy14 genome sequences (# 000001 to 063346) Click here for file

Additional file 3 **Supplement Table S5b: list of primers for 83,472 microsatellites found in *Cucumis sativus *Gy14 genome sequences (#063347 to 083472)**. Click here for file
